# Recent spectrophotometric and electroanalytical methods used for the determination of quinoline-based compounds

**DOI:** 10.1007/s43994-023-00051-8

**Published:** 2023-05-16

**Authors:** Hussain Alessa

**Affiliations:** Chemistry Department, Faculty of Applied Science, Umm Al-Qura University, Makkah, 21955 Saudi Arabia

**Keywords:** Anti-malaria, Quinoline compounds, Ultraviolet–visible spectrophotometry, Voltammetry, Square wave voltammetry, Differential pulse voltammetry

## Abstract

The current century experienced many disasters affecting the human-being existence. Malaria and new corona virus (COVID-19) are two deadly infections according to the world health organization (WHO). Different types of drugs were used for their treatment, for example quinoline-based drugs. The determination of these compounds in human body or pharmaceutical tablets is crucial for assessing the quality assurance during its manufacture, also for the medication trials. This review provides the current spectrophotometric and electroanalytical methods utilized for the determination of quinoline-associated compounds, such as chloroquine, hydroxychloroquine, quinine, mefloquine, piperaquine, primaquine and amodiaquine.

## Introduction

The world experienced disastrous diseases in the recent century like malaria and Corona virus (COVID-19) which had negative impact on the life of millions of people [1]. As for malaria, it is a disease disseminates to people by the bite of *Anopheles mosquitoes* female mosquito. There are different types of this mosquito, among which, the deadliest is *P. falciparum*. The current studies by the world health organization (WHO) estimated about 250 million infected people in 2021 with about 620 thousand death cases [2]. As malaria lives on the hemoglobin in blood, the disturbance of their blood cycle vanishes them, this is achieved by the intra-parasitic cumulation of heme by the suppression of heme polymerization, which is the mechanism by which these compounds have been used for treating malaria patient [3].

When it comes to COVID-19, its transfer and effect were not similar to malaria but its spread was faster and mainly affect the respiratory system, especially at people who are elderly or have certain diseases. Efforts were made worldwide to overcome these diseases and mitigate their effect. At early stages, different medicines associated with quinoline, such as chloroquine (CQ), hydroxychloroquine (HCQ), quinine (Q), mefloquine (MQ), piperaquine (PiQ), primaquine (PrQ) and amodiaquine (AQ) were prescribed for malaria treatments [4–12]. It should be mentioned that, some studies illustrated the death rates of COVID-19 patients increased after using these some of these medications, therefore they are prevented from use by the world health organization (WHO). The increase in this rate may be due to counterfeit or substandard drugs [13, 14].

**Fig. 1:**
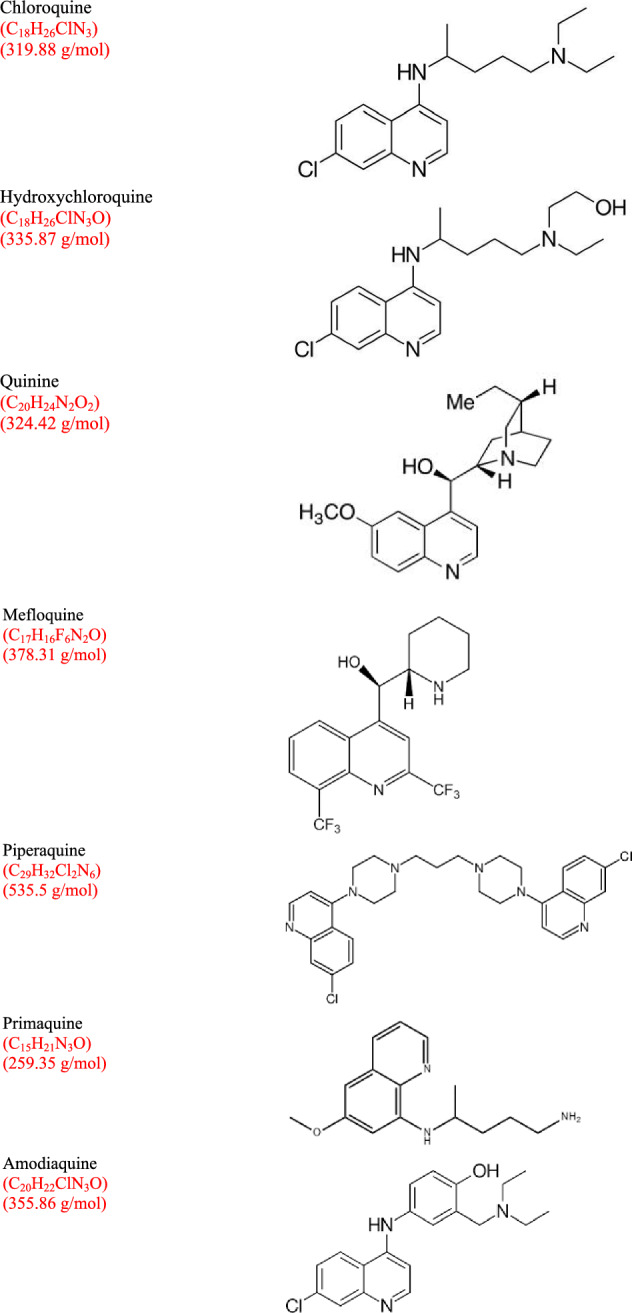
The structures, chemical formulas and molecular weights of some quinoline-based compounds

Some information regarding the structures, chemical formula and molecular weights of these compounds is shown in Fig. 1. Hydroxyl group differentiates HCQ from CQ. Both CQ and HCQ are effective against malaria, but the toxicity of CQ is higher than HCQ if used with small amounts for long time [1]. HCQ is safer but less effective compared to CQ. Many bacterial, viral, fungal infections and autoimmune diseases were mitigated by using CQ [15–19].

When considering Fig. 1, different quinoline-based compounds have different formulas and molecular weights, so they would have different physio-chemical properties like water solubility (S_w_) and octanol-H_2_O partitioning coefficient (K_ow_). The capability of these compounds to dissolve in lipophilic lipo-philic or hydrophilic solvents is affected by S_w_ and K_ow_ values. The reported values of pK_a_ of chloroquine, piperaquine and quinine were 10.1, 5.72 and 5.1, respectively. Although piperaquine is poorly soluble in water, other drugs are soluble, like CQ (0.01 g/L), PrQ (˃ 0.03 g/L) and Q (0.5 g/L) [20, 21].

The spectrophotometric properties, such as the molar absorptivity and Sandell's sensitivity, of the studied chloroquine compounds were extracted from the literature. These properties differ based on the compound, the solvent and the analysis method. For example, CQ phosphate could form ion-pairs by reacting with either Bromocresol Green dye and Bromocresol Purple dyes, the absorbance of these colored ion-pairs can be measured spectrophotometrically at 420 nm. CQ-PO_4_ had Sandell's sensitivity of 17.9 × 10^–3^ µg/cm^2^ and 10.4 × 10^–3^ µg/cm^2^ when reacted with the green dye and purple dye, respectively [22]. The molar absorptivity was 17.9 × 10^3^ L/mol.cm and 30.9 × 10^3^ L/mol.cm with green and purple dyes, respectively [22]. CQ diphosphate could also form ion-pair complex with bromocresol purple, the absorbance measurement at 420 nm resulted in Sandell's sensitivity of 12.6 × 10^–3^ µg/cm^2^ [23].

Some of electronic properties of CQ, CQ phosphate and HCQ were evaluated by the density functional theory (DFT), shown in Table 1, which has a correlation with their properties and effect [24, 25]. These were the energy of the highest occupied molecular orbital (HOMO), energy of the lowest unoccupied molecular orbital (LUMO), energy band gap, electron affinity, ionization potential and electronegativity values. The band gap, difference between the energies of LUMO and HOMO, provides insights about the conductivity nature of the compound and its stability under different radiations as well as its ability towards electrons transfer [24, 25].

**Table 1:** Some theoretically electrical parameters calculated by the density functional theory for chloroquine, chloroquine phosphate and hydroxychloroquine

	CQ	CQ phosphate	HCQ
HOMO (eV)	−5.594	−5.228	−5.517
LUMO (eV)	−1.115	−2.599	−1.22
Band gap (eV)	4.479	2.629	4.297
Electron affinity (eV)	1.115	2.599	1.220
Ionization potential (eV)	5.594	5.228	5.517
Electronegativity	3.354	3.913	3.369
References	[24]	[24]	[25]

When considering the analytical methods used for the isolation and quantification of quinoline-based compounds, they were mainly chromatographic, spectrophotometric and electrochemical ones. The first method can offer wide ranges of samples to be analyzed, but these methods have some disadvantages, such as they are the most expensive, they require tedious maintenance, they are relatively slow and require specialist for running the samples and explaining the results.

On the other hand, spectrophotometric and electroanalytical methods are relatively simple, easy to use and are cost-effective. Spectrophotometric methods require the samples to be colored which is not mandatory for electrochemical methods. The analytes can be detected at very lower concentrations with wider concentration ranges by electrochemical methods contrary to the spectrophotometry. An advantage of spectrophotometric methods would be from the ability to detect more than one drug in a simultaneous determination, whereas electrochemical methods can follow the redox reaction of one analyte at a time.

Tremendous amount of work has been documented in the literature regarding the determination, quantification and pharma-kinetics of these compounds. This work aims at summarizing the outcomes of various analytical methods, especially spectrophotometric and electroanalytical ones, used for the detection of these quinoline-based compounds in various samples' types, especially in the last 10 years.

## Reported sample preparation methods

In order to provide reliable results regarding the evaluation of the amounts of quinoline-based compounds in biological samples, the samples need to be well-prepared to remove the possible interfering molecules. Regarding this, some methods were reported in the literature, such as solid-phase extraction (SPE), liquid-phase extraction (LLE), supported liquid extraction (SLE), dispersive liquid–liquid microextraction (DLLME) and protein precipitation. All these methods are not simple, time-consuming with low selectivity. These anti-malaria compounds were detected by many analytical methods, such as spectrophotometric, electroanalytical and chromatographic ones. The last two methods were massively employed compared with the first one. Table 2 showed some of the methods used for the extraction of different quinoline-based drugs from different matrix (blood, urine, plasma, pharmaceutical tablets).

**Table 2:** Selected extraction methods reported in the literature regarding quinoline-based compounds

Analyte	Matrix	Extraction method	Linear range (µg/L)	LOD (µg/L)	LOQ (µg/L)	References
CQ	Water	SPE	0.1–10	0.2	0.8	[26]
PiQ	Human plasma	LLE	10–1000	3	10	[27]
Q	Urine	Ultrasound-assisted DLLME	NR	5.4	18	[28]
PrQ enantiomers	Human plasma	Protein precipitation and SPE	1.14–519	0.286 and 0.571	1.22 and 2.44	[29]
AQ	Human plasma	SLE	1.08–263	NR	1.08	[30]

As for SPE, it is one of the simplest preparation techniques for extracting, clean-up and preconcentrating the analytes before their detection. It is suitable for water, blood, urine and food matrix. The SPE rely on using cartridges having sorbents with different affinities towards various analytes in a sample. Usually, the cartridge is washed with deionized water, conditioned with a suitable solvent for wetting the sorbent. Then, a solution containing analyte and matrix is loaded onto the device, the sorbent would retain the analyte or impurities from the sample. Finally, the analyte or impurities would be eluted from the sorbent [26, 31, 32].

As for LLE, two immiscible solvents, aqueous and organic, having different polarities are used. The analyte would partition based on its affinity towards them. Usually, the mixture, sample solution and the two different solvents, is shaken for a specific time in a separatory funnel or a test tube. The transfer of the analyte to its favorable solvent may require single or multiple extractions. Then the two phases can be eluted separately, and the phases containing the analyte can be added together. If the target analyte is eluted with the organic solvent, the medium must be passed over a drying agent to eliminate possible water droplets that may exist in the organic phase. The final solution is then undergoing direct analysis, dilution if the analyte concentration is high, or if the chromatographic method is to be used for isolation and quantification, the eluted organic solvent may be dry by evaporation, then the residue would be dissolved in a volume of the mobile phase before administrating the sample to HPLC system. Although LLE is cheaper and more efficient than SPE, its selectivity is low, it also requires further purification steps if pure product is needed and it consumes larger solvent volumes that elevate the cost and rise environmental concern [27, 32].

With regard to SLE use a cartridge or well-plates (extraction columns) with mixed principles of SPE and LLE. The solid support, fine porous particles, interacts with the analytes and impurities in an aqueous sample, upon loading them onto SLE particles surface, in an adsorption process. These species are retained by the solid support, then an organic solvent is employed for eluting the target analyte whereas interferants would still adsorb on the support [30]. It is preferable over SPE and LLE, however, the extraction columns are of single-use, hence rising environmental concern and increasing the analysis cost.

Different approaches utilized protein precipitation (PP) method whether as a single preparation step or in coupled with SPE. When used alone, the organic solvent, like CH_3_OH or CH_3_CN, were added to the sample. The internal standards can be added to the either the sample or the solvent before forming the mixture. Then, the mixture is stirred well, centrifuged and a volume of the supernatant is injected into the analytical apparatus or evaporated then reconstituted [29, 33]. About 0.3 mL methanolic solution having internal standard was used during protein precipitation of 0.05 mL of plasma sample containing PiQ [34]. Also, 0.2 mL of cold methanol was used for the protein precipitation of 0.1 mL plasma that contained Q [35]. When looking at the literature, SPE and LLE can be used for the majority of matrix whereas PP is mainly utilized for plasma and blood samples and usually associated with chromatographic methods.

### Detection methods

Different analytical methods have been used for the analysis of anti-malaria compounds, particularly the ones mentioned in this work, such as spectrophotometric and electrochemical methods. A brief explanation of these methods will be presented with expressing their validity parameters, such as linearity range, recoveries, limit of detection (LOD) and limit of quantification (LOQ). As they are instrumental-based methods, calibration curves are mandatory to study the relationship between different standard concentration solutions, of the studied anti-malaria drugs, and the instrument physical property, which resulted in showing the linearity on the calibration curve. LOD and LOQ are calculated by multiplying the signal-to-noise ratio of each analytical method by 3 and 10, respectively. The validation of the reported methods was usually in accordance with the US pharmacopeia and the ICH guideline on validation of analytical procedures [36, 37].

### Spectrophotometric methods

As for spectrophotometric methods, the detection of the analytes is based on their interaction with the light parts produced by the light sources in the UV–Vis spectrophotometer; ultraviolet (UV), visible (Vis) and infrared (IR) parts of the light. [38]. It was observed that this type of compounds was seen to have the maximum absorption in the UV region, and usually spectrophotometric detectors like UV or diode array detector (DAD) are linked to HPLC for the detection of these compounds after being separated by the chromatographic methods. A summary of some spectrophotometric methods presented in the literature regarding the determination of quinoline drugs is shown in Table 3.

**Table 3:** Some of the selected spectrophotometric methods used for the detection of quinoline-based compounds

Analyte	Sample	Detection method	Wavelength max (nm)	Linear range (µg/L)	LOD (µg/L)	References
CQ and pyrimethamine	Pharmaceutical preparations	UV spectrophotometry	NR	20 × 10^2^–42.0 × 10^3^	NR	[39]
CQ	Plasma	Fluorometric	355	25 × 10^3^–60 × 10^4^	NR	[40]
CQ	Pharmaceutical preparations	Colorimetry	528	10 × 10^3^–30 × 10^3^	NR	[41]
CQ diphosphate	Urine and pharmaceuticals	UV–Vis spectrophotometry	420	1.25 × 10^3^–8.75 × 10^3^	NR	[23]
CQ	Blood and plasma	UV spectrophotometry	342	2.5 × 10^3^–25 × 10^3^	0.39 × 10^3^	[42]
CQ	Pharmaceutical preparations	UV spectrophotometry	343	10.88 × 10^3^–30.56 × 10^3^	NR	[43]
CQ diphosphate	Tablets	UV–Vis spectrophotometry	343	7.2 × 10^3^–19.2 × 10^3^	NR	[44]
CQ phosphate	Biological samples	UV–Vis spectrophotometry	NR	0.045–7.32 µM	6.88 nM	[45]
CQ phosphate	Pharmaceutical preparations	UV–Vis spectrophotometry (Bromocresol Green)	420	1 × 10^3^–20 × 10^3^	0.27 × 10^3^	[22]
CQ phosphate	Pharmaceutical preparations	UV–Vis spectrophotometry (Bromocresol Purple)	420	0.5 × 10^3^–12 × 10^3^	0.15 × 10^3^	[22]
HCQ	Pharmaceutical preparations	UV–Vis spectrophotometry	343	NR	0.1 × 10^3^	[31]
HCQ	Pharmaceutical preparations	UV spectrophotometry	343	1 × 10^3^–20 × 10^3^	NR	[46]
HCQ sulphate	Dosage form	UV–Vis spectrophotometry	220, 234, 256, 330 and 342	0.35 × 10^3^–0.55 × 10^3^	0.38 × 10^3^	[47]
Q	Biological samples	UV–Vis spectrophotometry	NR	25–700	14.71	[48]
MQ	Pharmaceutical formulation	UV–Vis spectrophotometry	222	20 × 10^3^–120 × 10^3^	0.45 × 10^3^	[49]
AQ HCl	Tablets	UV–Vis spectrophotometry (Zero and first order derivatives)	217.5 and 343	10 × 10^3^–70 × 10^3^	0.032 × 10^3^ and 2.716 × 10^3^	[50]
AQ	Dosage form	UV–Vis spectrophotometry	275	10 × 10^3^–60 × 10^3^	0.825 × 10^3^	[51]
PrQ	Tablets	UV–Vis spectrophotometry	NR	10 × 10^–3^–60 × 10^–3^	3.2 × 10^–3^	[52]

The analysis of the quinoline-based compounds is usually performed by using a double-beam spectrophotometer for measuring their absorbance in different media. One of the requirements is choosing a suitable solvent for dissolving each compound without causing instability to the drugs during the course of analysis. Some solvents were reported to fulfil this criterion, such as distilled water, hydrochloric acid and sulfuric acid. A standard calibration curve must be established, this is by preparing different concentrations of the drug and scan their absorption at a range of wavelengths. Then extracting the highest absorbance value, at noted wavelength known as maximum wavelength (λ_max_), which is normally in the UV region; below 400 nm. Beer's law must be obeyed and the optimized calibration curve, which show a strong correlation between the concentration of the target analyte and their absorbance, would have a straight-line equation; y = ax ± b, in which y is the absorbance value, x denoted the analyte concentration, a is the slope whereas b is the y-intercept, can be used to determine the unknown concentration in the studied sample.

When using UV–Vis spectrophotometry, most reported analyzed samples are pharmaceutical tablets or spiked urine samples. Tablets are normally weight, crushed and homogenously powdered by using a pestle and a mortar. This was followed by weighing a certain amount of the powder, dissolving in a suitable solvent then filtrating the solution by using Whatman paper, a volume of the filtrate would be treated by SPE or LLE for extracting the analyte of interest. The final solution after the extraction may contain small volume of the extraction solvent like chloroform, hence heating the solution in the presence of HCl may be required to remove the extraction solvent [43].

In the olden days, Khalil et al. determined CQ by spectrophotometric method that based on forming ion-pair complex with Mo (V). Ascorbic acid was used for reducing Mo (IV) to Mo (V) that was then treated with ammonium thiocyanate to for a complex of red color. The presence of CQ in the sample would react with the complex which changed the color to orange-red [39].

Later on, CQ was quantified in human plasma samples by fluorescent method, in which CQ was derivatized electrochemically before being spectrophotometrically detected [40]. Samanidou et al. determined CQ and Q simultaneously by using fluorescent detector attached to RP-HPLC [31]. The analytes were extracted by SPE from biological samples and pharmaceutical preparations. They were eluted using isocratic elution using a mixture of methanol and acetonitrile as a mobile phase. The excitation wavelength (λ_exi_) and emission wavelength (λ_emi_) were set at 325 and 375 nm, respectively [31].

Also, CQ was quantified in pharmaceuticals using HCl as a solvent at 342 nm, the absorption maximum (λ_max_). High linear range was observed when applying Beer's law. The molar absorptivity was 0.0089 L.mol^−1^ cm^−1^. The method determined CQ with recoveries up to 100% [42].

To overcome the health-related problems associated with the organic solvents used in spectrophotometric measurements, Desta and Amare reported the use of distilled water instead [43]. Their approach was used when detecting CQ phosphate in tablets in which the λ_max_ was observed at 343 nm. Beers' law was obeyed and linearity was noticed in mM concentration range [43]. Also, Singh et al. developed a spectrophotometric method for quantifying HCQ sulphate in pharmaceutical preparations. Hydrochloric acid (HCl) was employed as a solvent and 343 nm was set as the λ_max_. The method exhibited HCQ linear response over nM concentration range [46].

Q was quantified in pharmaceutical preparations using a complex consisted of *o*-sulfophenylfluorone, Q and Cu (II). Linearity was observed with acceptable validation parameters [53]. Moreover, fluorometric method was reported to be suitable for quantifying Q in pharmaceutical tablets and non-alcohol-added drinks. H_2_SO_4_ was employed for the study of Q luminescence which produced very small noise. Linearity was noticed in 0.1 × 10^3^–1.0 × 10^3^ nM concentration range and LOD was calculated to be 2.9 µM [54]. The Q tablets distributed in Congo republic was examined by Namegabe et al. The qualitative analysis was conducted by spectrophotometric analysis, in which tablets were dissolved in deionized water, then treated with concentrated H_2_SO_4_, left in the dark and experienced UV light at 366 nm which produced white blue florescent, this light is a fingerprint confirming Q presence [55].

As the bitter taste of soft drinks can be enhanced by the addition of Q. Q was studied in some drinks by 3^rd^ derivative spectrophotometry at 578 nm and quantified at 320 nm [44]. Also, Q was found in tonic water and determined spectrophotometrically at 347.5 nm, with LOD around 35 mg/L [56].

The treatment of PrQ with 1,2-Naphthoquinone-4-Sulfonate yielded brown color in basic medium, pH of 10, having λ_max_ at 485 nm. This facilitated its quantitation in tablets by spectrophotometry [52].

Both CQ and AQ were simultaneously determined by spectrophotometry. They were oxidized by KBrO_3_ and KIO_3_ and the process was studied at 342 nm and 343 nm. They were quantified in pharmaceuticals. The method showed linearity in 0.5 × 10^3^–50 × 10^3^ µM and 0.2 × 10^3^–4.0 × 10^3^ µM for CQ and AQ, respectively, with LODs of 0.06 × 10^3^ µM and 0.4 × 10^3^ µM for the analytes [57].

### Electroanalytical methods

The most used electroanalytical methods for the analysis of quinoline-associated compounds are based on voltammetry and potentiometry. As for voltammetry, a fixed or varied potential is applied at the working electrode to cause oxidation or reduction of the analyte species. This redox process changes the analyte concentration at the working electrode. The electrochemical studies are conducted in a medium that consisted of electrolyte support. The physical properties, for examples the solubility, electrical conductivity and the electrochemical reactivity, of the studied drug influenced the used medium. The electrolyte must not react with the analyte or the product as well as it should reduce the solution resistance [58, 59].

Some electroanalytical methods like cyclic voltammetry (CV), differential pulse voltammetry (DPV), linear sweep voltammetry (LSV), square wave voltammetry (SWV), stripping voltammetry, adsorptive stripping differential pulse voltammetry (AdSDPV) and amperometry methods were reported for the detection of quinoline-associated compounds in various samples. The electroanalytical measurements were done on carbon paste electrode (CPE), glassy carbon electrode (GCE), poly vinyl chloride (PVC), boron-doped diamond electrode (BDD).

The electroanalytical methods have some merits, such as simplicity, higher selectivity, stability with high reproducibility, easily sample preparation, large linearity range and consume less amount of solvent, over the other analytical methods [60, 61]. The performance of these methods is affected by the working electrode type. As would be seen, modified electrodes have better performance, enhanced sensitivity with low LOD over unmodified ones.

The literature showed DPV as the most used electroanalytical method. It is a derivative of LSV or staircase voltammetry. During its use, a chain of voltage pulses is superimposed onto raised potential of stairsteps or linear sweep voltage. The resulted current is detected just before or after each pulse [62]. In CV, the potential of the working electrode is applied in forward and backward directions while the current is measured for each direction. Anodic current and cathodic current for oxidation and reduction of the analyte, respectively. The difference in the current between each direction is measured for SWV [63].

In relation to potentiometry, an electrochemical system having two or three electrodes is used. The analytes can be quantified by measuring the potential difference between two electrodes; working and reference electrodes. A summary of some electroanalytical methods presented in the literature regarding the determination of quinoline drugs is shown in Table 4.

**Table 4:** Selected electroanalytical methods used for the detection of quinoline-based compounds

Analyte	Detection method	Electrode	Sample	Linear range (µg/L)	Detection limit (µg/L)	References
Q	Potentiometric	Modified PVC membrane	Soft drinks	3.24 × 10^5^–12.98–10 × 10^5^	0.004 and 2.66	[64]
Q	SWV	Modified GCE	Commercial injection samples	0.973 × 10^3^–32.44 × 10^3^	0.142 × 10^3^	[65]
Q	CV	Polypyrrole-pentacyanoferrate/Platinum (PPY–PCNFe/Pt) electrode	Aqueous solutions	3.24 × 10^5^–29.19 × 10^5^	3.50 × 10^3^	[66]
Q	SWV	Modified GCE	Human urine and pharmaceutical formulation	3.24 × 10^3^–32.44	4.54	[67]
Q	SWV	Hanging mercury drop electrode	Surfactant media	9.73 × 10^3^–74.62 × 10^3^	0.04 × 10^3^	[68]
Q	Amperometry	Modified GCE	Tonic water	0.26 × 10^3^–84.34 × 10^3^	6.48	[69]
Q	DPV	Pencil graphite electrode	Soft drinks	0.16 × 10^3^–32.44 × 10^3^	64.88	[70]
Q	DPV	Molecularly imprinted composite sensor	Plasma and urine samples	0.32 × 10^–3^–0.32	1.62 × 10^–5^	[71]
Q	Potentiometric	Modified ion-selective electrode	Human urine	0.78 × 10^3^–32.44 × 10^5^	0.20 × 10^3^	[72]
CQ	DPV	Modified carbon paste electrode	Serum	31.98–31.98 × 10^2^	9.59	[73]
CQ	DPV	Modified carbon paste electrode	Pharmaceutical tablets	21.75 × 10^6^–22.00 × 10^8^	31.98 × 10^5^	[74]
CQ	DPV	Modified GCE	Human serum and pharmaceutical formulations	0.16 × 10^3^–26.36 × 10^3^	12.79	[75]
CQ	SWV	BDD	NR	3.19–79.97	0.64	[65]
CQ	DPV	3D-printed polylactic acid stencil	NR	1.59 × 10^3^–23.99 × 10^3^	1.28 × 10^3^	[76]
CQ phosphate	Potentiometric	Modified CPE	Tablets	51.59–12.02 × 10^4^	5.16	[77]
CQ phosphate	Potentiometric	Modified anode and modified cathode	Serum and water	5.16 × 10^3^–51.59 × 10^4^	56.74	[78]
CQ phosphate	SWV	Composite of rGO and Cu NPs	Tap water	0.26 × 10^3^–56.74 × 10^3^	0.12 × 10^3^	[79]
MQ HCl	DPV	Hanging mercury drop electrode	Tablets and biological fluids	2.48 × 10^3^–33.18 × 10^3^	0.18 × 10^3^	[80]
MQ	SWV	Hanging mercury drop electrode	Tablets and biological fluids	NR	98.74	[80]
MQ	SWV	Modified GCE	Human urine and pharmaceuticals	0.38 × 10^3^–37.83 × 10^4^	0.53	[81]
PrQ	LSV	GCE	Commercial tablets	7.78 × 10^3^–25.94 × 10^5^	9.38 × 10^3^	[82]
PrQ	DPV	GCE	Commercial tablets	7.78 × 10^3^–25.94 × 10^3^	4.20 × 10^3^	[82]
PrQ	SWV	GCE	Commercial tablets	7.78 × 10^3^–25.94 × 10^3^	17.99 × 10^2^	[82]
PrQ	DPV	Modified carbon paste electrode	Pharmaceutical tablets	0.58 × 10^3^–5.89 × 10^3^	0.25 × 10^3^	[74]
PrQ	Differential pulse anodic stripping voltammetry	Modified pencil graphite electrode	Biological samples and pharmaceuticals	0.52–2.19 × 10^2^	2.08 × 10^2^	[83]
PrQ	DPV	Modified GCE	Tablets and human urine	2.59–259.35	0.91	[84]
PrQ	SWV	Modified GCE	Tablets and human urine	0.26–259.35	0.26	[84]
PrQ	SWV	Modified GCE	Urine	25.94–12.96 × 10^2^	7.26	[85]
PrQ	DPV	Modified GCE	Pharmaceuticals	51.87 × 10^2^–93.36 × 10^2^	0.52	[86]
AQ	Potentiometric	Poly(vinyl chloride) (PVC) membrane sensors	Pharmaceutical forms	11.38 × 10^2^–71.17 × 10^5^	NR	[87]
AQ	SWV	Hemin biosensor	Maternal milk	6.76 × 10^3^–35.58 × 10^3^	25.05 × 10^2^	[88]
AQ	DPV	Modified GCE	Pharmaceuticals and human urine	35.58–12.46 × 10^2^	31.67	[89]
AQ	DPV	Pencil graphite electrode	Various samples	0.36–71.17	0.10	[90]
HCQ	AdSDPV	Modified CPE	Biological and pharmaceutical samples	19.14–33.58 × 10^3^	2.02	[91]
HCQ sulphate	Potentiometric	Modified PVC membrane	Tablets	40.36 × 10^3^–43.39 × 10^5^	20.39 × 10^3^	[92]
HCQ sulphate		Modified CPE	Urine and tablets	4.34–30.02 × 10^2^	0.06	[93]
HCQ	DPV	Modified Au electrode	Pharmaceuticals	NR	28.54	[94]
HCQ	DPV	Modified CPE	Wastewater and human urine	0.13 × 10^3^–13.43 × 10^2^	10.74	[95]
HCQ	DPV	Modified GCE	Human fluid	30.22–34.29 × 10^2^	1.65	[96]
HCQ	SWV	Modified boron-doped diamond (BDD)	Tablets and urine	33.58–638.15	20.15	[97]
HCQ	DPV	Cork-graphite	River water	1.68 × 10^3^–21.83 × 10^3^	0.35 × 10^3^	[98]
HCQ	DPV	Cork-graphite	River water	83.96 × 10^4^–13.43 × 10^6^	49.04 × 10^4^	[99]
HCQ	SWV	3D-printed sensor	NR	0.13 × 10^3^–2.52 × 10^3^	13.43	[100]

Kamel and Sayour developed a potentiometric sensor for quantifying Q in soft drinks. The membrane was attached with molecular imprinted polymer (MIP). Q was determined in weak acidic medium. The improved sensor exhibited wider linearity range and LODs as low as 1.2 × 10^–6^ mM [64].

The electrocatalytic oxidation of Q at GCE was studied, it was found that the modification of GCE by using a gel having multiwall carbon nanotubes (MWCNTs) and 1-Butyl-3-methylimidazolium hexafluorophosphate (as ionic liquid), and conducting the work using a phosphate buffer of pH 6.8, would result in revealing the electrode mechanism to be controlled by diffusion and two electrons were involved in the oxidation process [65]. Both CV and SWV were used to study the electrochemical behavior of Q. The use of Britton–Robinson buffers of pH 10.38 with the addition of 1% CTAB was proved to enhance the signal of reduction peak current of Q. It is then postulated that the nitrogen of the Q moiety would have experienced a protonation process [68].

It was determined that, the detection of Q is improved when the working electrodes are modified. For example, Geto et al. reported improved determination of Q by modifying GCE with 4-amino-3-hydroxynaphthalene sulfonic acid. The modification enhanced the LOD as lower as 1.42 × 10^–2^ µM with recoveries higher than 90% [67]. Also, polymerization process would modify GCEs which resulted in lowering the LOD to 10^–2^ µM when detecting Q in tonic water [69]. Another method for improving the detection is by coating the working electrode by a composite. An acidic solution spiked with HAuCl_4_ during the electrodeposition of 3-methyl-4-nitrophenol and L-tyrosine was reported. This was to incorporate gold nanoparticles onto the created composite. The composite was used for determining Q in some biological samples [71].

Furthermore, a potentiometric sensor was fabricated for the detection of Q in human urine, in which the ion-selective electrode (ISE) was doped with melanin (as ionophore). The doping enhanced the selectivity and showed no interference from similar molecules like Q and HCQ [72].

Pristine and modified CPE were used for studying the electrochemical characteristic of CQ [73]. The systematic study over a range of buffer solutions and different pH by using CV and DPV. It showed that one irreversible oxidation peak appears when using pH 2.0–11.0 whereas another peak could be seen when using buffers with pH 5.0–7.0. The modification of the electrodes with DNA improved the sensitivity and enhanced the LOD and the recoveries [73]. Also, the modification of CPE with copper hydroxide, Cu(OH)_2_, nanowires improved the electrode sensitivity and lower the LOD [74].

Owing to its high surface area and higher conductivity, gold nanourchins were drop-coated onto GCEs to modify them. This was proven in the electrode performance; 0.002–1.0 µM as linear range with LOD as 1.4 nM [81]. Another modifying agents were graphene oxide and tungsten disulfide quantum dots, the modified GCE was used for the determination of CQ in human serum and medical preparations, by employing CV and DPV, better results were obtained such as wide linearity and LOD of 40 nM [75].

Oliveira et al. found that the irreversibility of the CQ anodic response could be overcome by using anodic or cathodic pretreatments of BDD before using SWV. The cathodically-pretreated BDD electrode showed a better-appeared anodic peak with higher current signal [101]. Very recently, an electrochemical electrode was fabricated by mixing graphite with SnO_2_ nanoparticles to modify CPE, then used it for the determination of CQ phosphate. The composite electrode showed the detection ability in the nM concentration range with LOD of 10 nM [77]. Moreover, a sensor based on using WO_3_/TiO_2_ as a photoanode and (Au nanoparticles/C/MoS_2_) as a photocathode was employed for the determination of CQ phosphate in river water and serum. This structure enables the sensor to be self-operated [78].

MWCNTs coated onto CPE would increase the electrode conductivity and HCQ signal when using AdSDPV method for the determination of HCQ in biological and pharmaceutical samples. This modification resulted in quantifying HCQ down to 6.0 nM [91]. Silva et al. improved the performance of CPE for the determination of HCQ in urine and tablets by treating the electrode with carbon nitride nanosheets. The electrode showed high selectivity towards HCQ with minimal noise from interfering species [93]. Matrouf et al. studied the use of direct and alternating current (DC and AC) to exfoliate graphite pencil, then applied CV for the electrodeposition of graphene oxide onto CPE. When comparing the electrodes prepared by DC and AC for the quantification of HCQ in wastewater and human urine, the latter method showed better validation parameters [95].

A modified PVC membrane with graphite rod was developed by Khalil et al. and showed good performance when determining HCQ sulphate in its forms. This composition enhanced the selectivity, sensitivity and the linear dynamic range during the potentiometric measurement [92]. BDD electrode underwent cathodic pretreatment then detected HCQ in biological samples at sub micromolar concentrations [97]. Due to the consumption of HCQ for treating COVID-19, it may be released to water, hence its monitor is important, for this purpose a composite sensor based on cork-graphite was used for its analysis in river water. The oxidation process of HCQ in the water site resulted in its removal which confirmed by BDD electrodes [99].

A fluorescent sensor based on aptamer was developed for the determination of MQ and PiQ in serum collected from malaria-infected people as well as in tablets [102].

Arguelho et al. reported a direct determination of PrQ in commercial tablets without pre-treatment. They used GCE for detecting PrQ and employed LSV and DPV and SWV for the electrochemical measurements. LODs as low as 0.18 × 10^4^ µM were reported [82]. Modifying pencil graphite electrode (PGE) with fullerene (C_60_) prompted the electrode kinetic, as fullerene shuttles electrons between the recognition sites and PGE. As a result, the anodic current was enhanced by 5 folds with regard to unmodified one [83].

Thapliyal et al. showed that modifying GCEs with gold nanourchins improved its electron transfer, opposite to bare GCEs, which enhanced the electrocatalytic activity of oxidizing PrQ. The modified GCEs exhibit better validation parameters, with 10^–2^ µM LODs, of PrQ in urine samples when using SWV and DPV [84]. The trace amounts of PrQ in urine samples requires the use of LLE and TLC to enhance the selectivity and preconcentration of PrQ. These steps were accompanied by employing modified GCE with multi-walled carbon nanotubes. The modification of GCE was to enhanced its surface area for better electron transfer which was witnessed in lower LOD [85].

A sensor based on PVC membrane ion selective electrode (ISE) was used for detecting AQ hydrochloride [87]. Different sensors were fabricated using different plasticizers. They displayed good validation parameters; rapidity, selectivity, stability and near Nernstian response over AQ concentrations of 32 × 10^–7^–0.02 M in pH = 3.7–5.5 [87]. The use of hemin biosensor for electrochemical study of AQ produced a distinguished AQ oxidation peak at 0.14 V against silver/silver chloride electrode. AQ was detected in breast milk as low as µM range [88]. The modification of GCE by electrochemical polymerization of MWCNT and methyl orange was examined in detecting AQ in human urine and pharmaceutical formulations. The electrochemical oxidation of AQ was studied by CV and DPV [89]. A number of bare carbon-based electrodes were studied for AQ electrochemical characteristics. Among which, the highest oxidation current for AQ was obtained when using bare PGE. The DPV experiments on various samples resulted in 3.0 × 10^–4^ µM as AQ LOD with recoveries over 100% [90].

## Conclusion and future insights

The quinoline-based compounds have been employed for treating malaria patients. This work illustrates the use of spectrophotometric and electroanalytical methods for their determination in different matrix; biological, pharmaceuticals, herbs and drinks. A limited use of spectrophotometric methods is noticed in the literature, to the contrary higher publications regarding electroanalytical methods. The use of voltametric methods exceeded the potentiometric ones. The discussion of the types of the working electrodes is presented for all modified and unmodified electrodes. When comparing the two detection methods, electrochemical methods provide faster, more reliable, cost-effective and enable detection at very lower concentration ranges compared to the spectrophotometric ones. They also do not require samples to be colored, contrary to spectrophotometric methods which usually detect analytes at higher concentrations and have poor absorbance detection for transparent samples. More studies are required for the isolation and quantification of piperaquine and primaquine due to their very limited literature work. The majority of the used working electrodes were carbon-based, additional studies need to focus on using various metal oxides, 2D inorganic compounds (like MXenes), 3D-printed sensors and metal organic frameworks for modifying the present electrodes needs consideration. Additionally, the fabrication of optoelectronic sensors for on-site determination of these drugs would be of concern.

## Data Availability

The data will be made available on reasonable request.
